# Supplementary material for: A dsRNA virus with filamentous viral particles
Source: Nat Commun. 2017 Aug 1;8:168. doi: 10.1038/s41467-017-00237-9 (PMC5537263; doi:10.1038/s41467-017-00237-9)
Supplement: Supplementary file 1 — Supplementary Information [file 41467_2017_237_MOESM1_ESM.pdf]

File name: Supplementary Information
Description: Supplementary Figures, Supplementary Tables and Supplementary References

File name: Peer Review File
Description:

		IV		V		VI
		D.#.&D		G..SG...T.&.		GDD.&&
CcFV-1	query	DIAKWDANMCE	[54]	GCMPSGSYYTSML	[28]	TLISYGDNQLI
AfuTmV-1	CDP74618	DITKWDANMC	[55]	GCMPSGSFYTSLV	[42]	TLISYGDNQLF
BbPmV-1	LN896307	DIEKWDANMS	[55]	GCMPSGSFYTSLL	[42]	ALLSYGDNQLI
BbPmV-2	LN896318	DILKWDASVV	[55]	GTMPSGSYYTSLI	[42]	ALLSYGDNQLI
BdRV-1	AKE49495	DIEKWDANMC	[55]	GCMPSGSYYTSLI	[42]	TLVSYGDNQLI
RHDV	1KHV_A	DYSKWDSTMSP	[43]	RGLPSGMPFTSVI	[31]	PFYTYGDDGVY
HCV	1GX6_A	DTRCFDSTVTE	[42]	RCRASGVLTTSCG	[21]	TXLVNGDDLVV
BVDV	1S4F_A	DTKAWDTQVTS	[45]	GQRGSGQPDTSAG	[28]	RIHVCGDDGFL
RCNMV	gi 133643	DASRFDQHCSV	[47]	GCRMSGDINTGLG	[20]	SLANNGDDCVL
FCVC6	gi 6226677	DYSKWDSTQSP	[43]	SGLPSGMPLTSVI	[32]	DMMTYGDDGVY
B. cinicola	gi 15375092	DFSKFDSTIPP	[47]	GGNVSGSFLTSAL	[24]	DLFILGDDFIL
SPMMV	gi 25013881	DGSRFDSSIPP	[47]	RGNNSGQPSTVVD	[23]	KYVCNGDDLLI
AEV	gi 40538720	DFSNYDASLSP	[43]	GSMPSGTPATSVL	[28]	KILTYGDDVLF
CtRLV	gi 2582372	DASRFDLHVSV	[47]	GGRCSGDSDTSLG	[21]	EVANDGDDQVL

Supplementary Fig. 1 Three conserved motifs of deduced RNA-dependent RNA polymerase (RdRp) from *Colletotrichum camelliae* filamentous virus 1 (CcFV-1) and other RNA viruses or organisms corresponding to motifs IV, V, and VI, respectively, as revealed by BLASTp. The "#" symbol refers to residues S or T, and "&" denotes any bulky hydrophobic residue (L, I, V, M, F, Y, W). Numbers within the brackets indicate the number of amino acids not shown. Virus names and acronyms are listed as follows: RHDV (*Rabbit hemorrhagic disease virus*), HCV (*Hepatitis C virus*), BVDV (*Bovine viral diarrhea virus*), RCNMV (*Red clover necrotic mosaic virus*), FCVC6 (*Feline calicivirus* strain CFI/68 FIV), *B. cinicola* (*Bryopsis cinicola*), SPMMV (*Sweet potato mild mottle virus*), AEV (*Avian encephalomyelitis virus*), CtRLV (*Carrot red leaf luteovirus*-associated RNA), AfuTmV-1 (Aspergillus fumigatus tetramycovirus-1), BdRV-1 (Botryosphaeria dothidea virus 1), BbPmV-1 (Beauveria bassiana polymycovirus-1), BbPmV-2 (Beauveria bassiana polymycovirus-2).

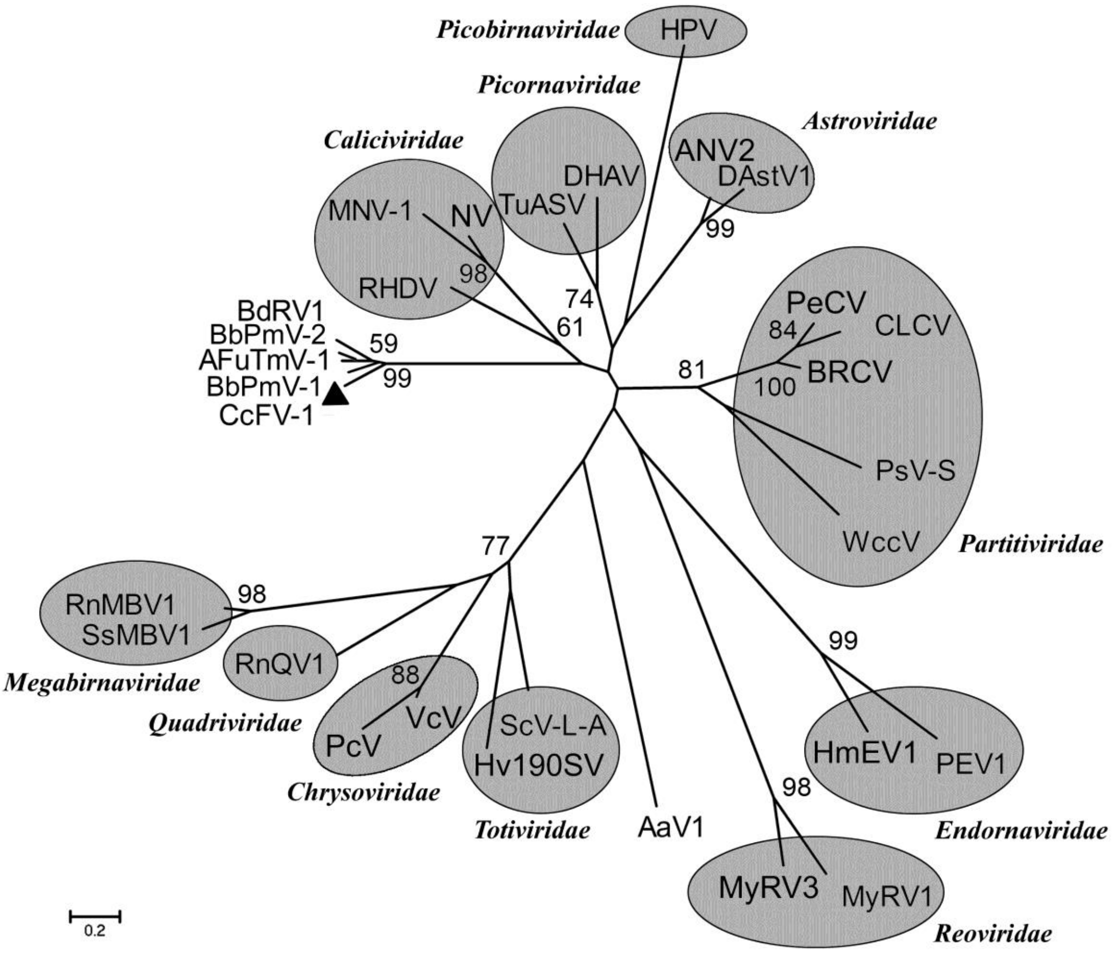

Supplementary Fig. 2 Phylogenetic analysis of the deduced RdRp sequences of CcFV-1 and selected members of all eight dsRNA virus families and unclassed dsRNA viruses belonging to group III and three (+)RNA virus families closer to CcFV-1, as listed in Supplementary Table 7. The phylogenetic trees were constructed as previously described [1].

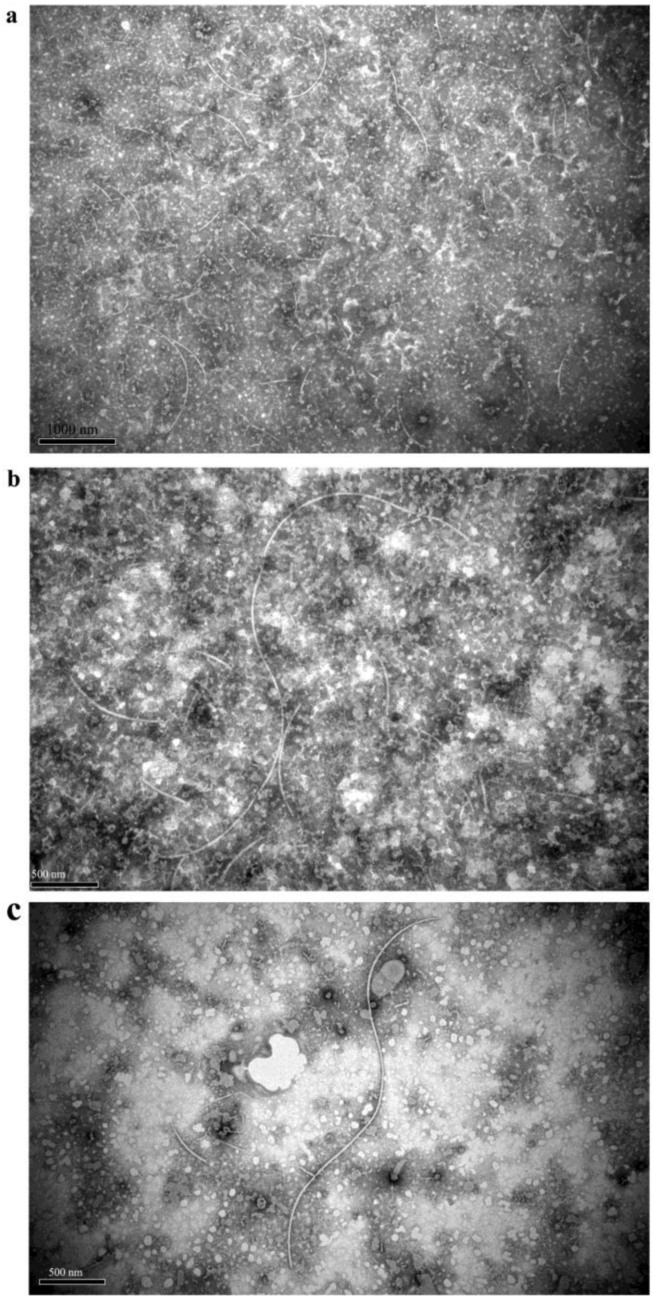

Supplementary Fig. 3 Transmission electron microscopy (TEM) image of virus-like particles extracted from *C. camelliae* strain LT-3-1. (a) Representative virus-like particles with filamentous particles longer than 127.1 nm were observed in the crude extract. Scale bar, 1000 nm. (b) Virus-like particles of 4426.7 nm in length and 12.4 nm in width (no. 6 in Fig. 32) observed in the 30% sucrose fraction. Scale bar, 500 nm. (c) The longest filamentous particles purified after centrifugation in 1.45 g/cm$_3$ CsCl. Scale bar, 500 nm.

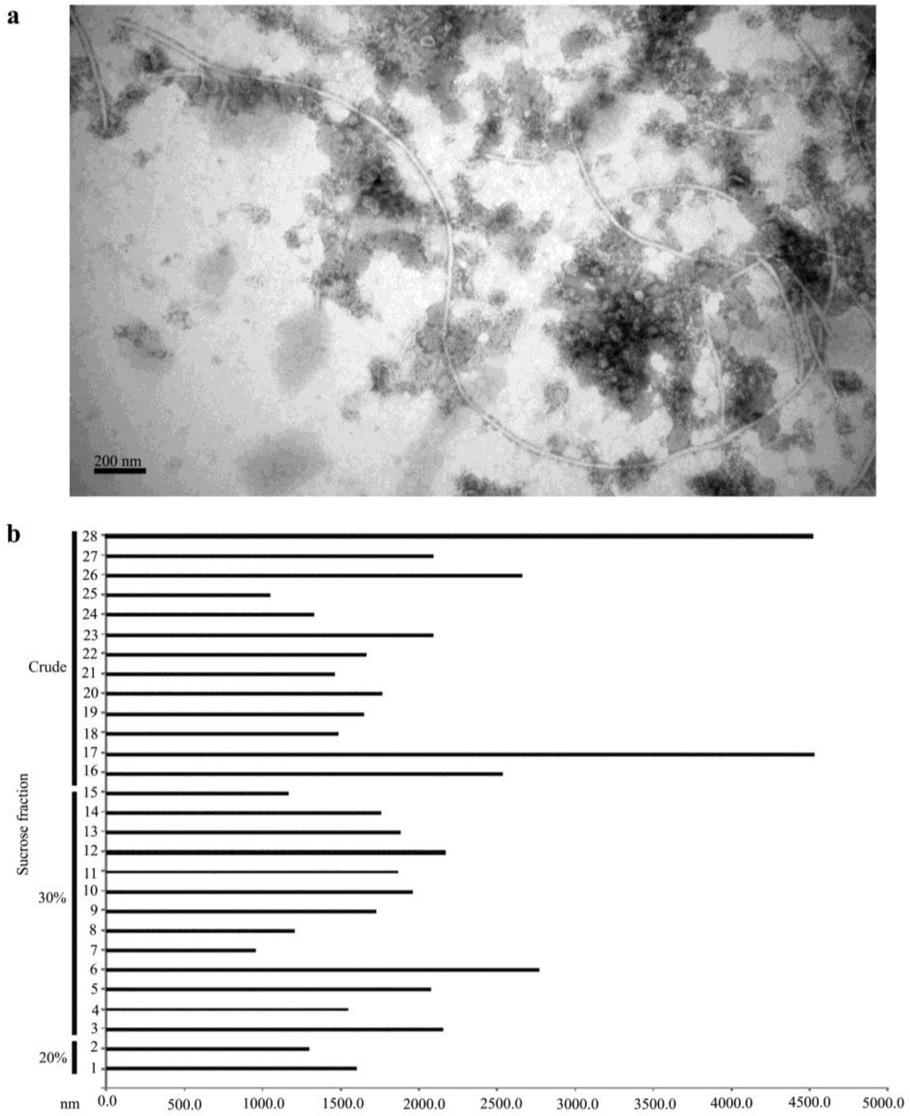

Supplementary Fig. 4 Immunosorbent electron microscopy (ISEM) images of virus-like particles decorated by PAb-P4 and a histogram of the sizes of decorating particles longer than 1000.0 nm. (a) The second longest virus-like particle in the crude extract decorated by PAb-P4 at an 8000-fold dilution observed by ISEM. Scale bar, 200 nm. (b) A histogram of the sizes of decorated particles longer than 1000.0 nm in the extracted LT-3-1 crude extract and in fractions corresponding to 30% and 20% sucrose, respectively, following sucrose gradient centrifugation. The numbers on the vertical axis indicate the numbers randomly assigned to virus-like particles.

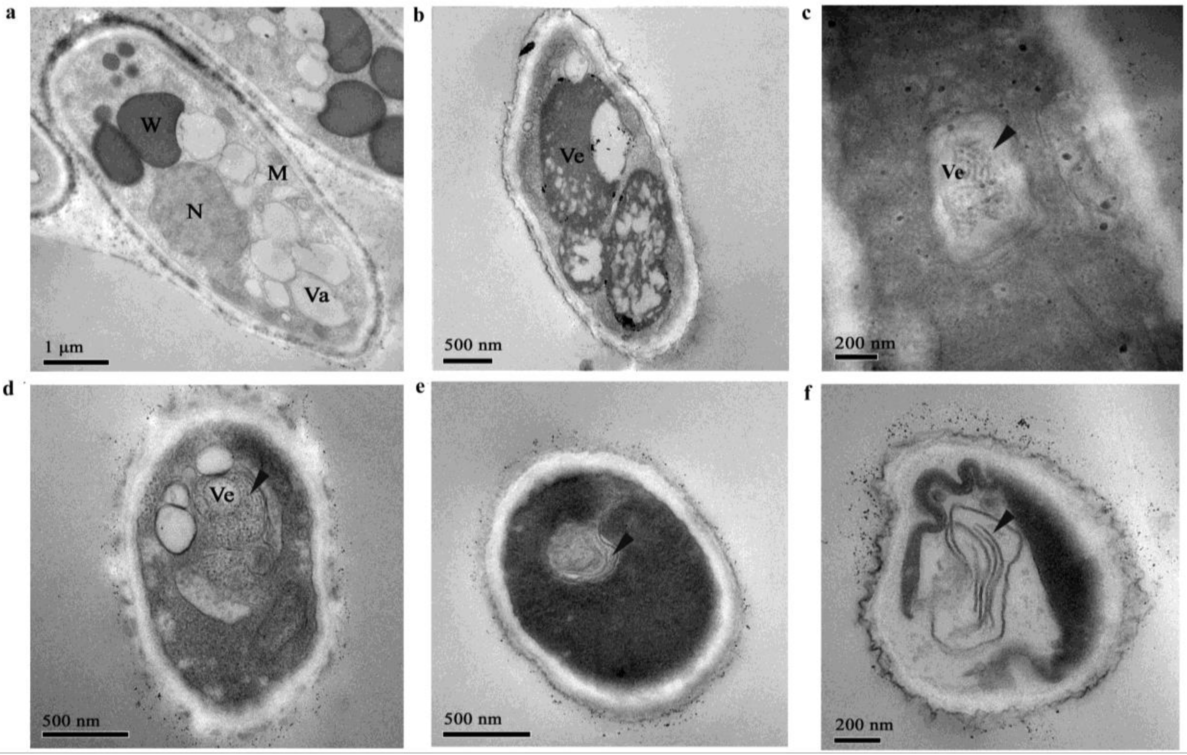

Supplementary Fig. 5 TEM images of ultrathin hyphal sections of *C. camelliae* strains LT-3-1 and LT-3-1D2. (a) Representative ultrathin hyphal section of strain LT-3-1D1 showing normal cellular structures. Scale bar, 1000 nm. (b) Representative ultrathin hyphal section of strain LT-3-1 showing degeneration. Scale bar, 500 nm. (c and d) Filamentous virus-like particles observed in the vesicles of strain LT-3-1 cells. The arrows indicate filamentous virus-like particles. Scale bars, 200 and 500 nm, respectively. (e and f) Unclear structures observed in the cytoplasm of hyphal cells of strain LT-3-1. Scale bars, 500 and 200 nm, respectively. N, nucleus; Va, vacuole; Ve, vesicle; M, mitochondrion; W, Woronin body.

Supplementary Table 1 Percent identity of the termini of dsRNAs 1 to 8.

	dsRNA1	dsRNA2	dsRNA4	dsRNA3	dsRNA5	dsRNA6	dsRNA8	dsRNA7
dsRNA1		76.5*	73.5	73.5	64.5	50	52.9	52.9
dsRNA2	33.7**		66.3	75.6	70.9	19.2	30.2	24.4
dsRNA4	21.1	24.3		63.4	68.8	26.7	37.8	32.6
dsRNA3	45.3	25	31.2		66.9	16.9	29.1	19.2
dsRNA5	31.7	25.4	23.8	47.6		19.1	36.4	23.3
dsRNA6	24.2	31.1	22.4	24	22.2		45.4	47.1
dsRNA8	28.4	19.4	24.4	28.1	30.2	22.1		47.7
dsRNA7	31.6	25.2	26.5	27.1	27	25.8	26.3	

* and **, percent identity of the 5- and 3-termini respectively.

Supplementary Table 2 Summary of the results of a BLASTp search for deduced proteins encoded by dsRNAs 1 to 8.

ORF No.	Species	Proteins	Total score	Query cover (%)	E value	Identity (%)	GenBank accession no.
1	Cladosporium cladosporioides virus 1	putative RNA-dependent RNA polymerase	741	99	0	53	YP_009052470
	Aspergillus fumigatus tetramycovirus-1	RNA-dependent RNA polymerase	522	84	7E-172	47	CDP74618
	Alternaria tenuissima virus	RNA-dependent RNA polymerase	508	70	9E-170	54	AJP08049
	Botryosphaeria dothidea virus 1	RNA-dependent RNA polymerase	504	84	4E-165	47	AKE49495
	Turkey astrovirus	RNA-dependent RNA polymerase	43.5	25	1.2	25	ABX46574
	Avastrovirus 3	RNA-dependent RNA polymerase	42.7	22	2.2	23	ABX46565
	Turkey astrovirus 2	RNA-dependent RNA polymerase	40.8	21	5.2	25	AFG33157
2	Cladosporium cladosporioides virus 1	hypothetical protein	514	99	6E-171	46	YP_009052471
	Aspergillus fumigatus tetramycovirus-1	hypothetical protein	232	94	4E-63	32	CDP74619
	Alternaria sp. FA0703	hypothetical protein	211	92	1E-55	31	ACL80752
	Botryosphaeria dothidea virus 1	hypothetical protein	202	92	2E-52	32	AKE49496
3	Cladosporium cladosporioides virus 1	hypothetical protein	491	89	5E-164	47	YP_009052472
	Aspergillus fumigatus tetramycovirus-1	Methyl transferase	140	85	1E-31	26	CDP74620
	Botryosphaeria dothidea virus 1	hypothetical protein	127	74	1E-27	27	AKE49497
	Thermovenabulum gondwanense	Polyamine aminopropyltransferase	48.5	19	0.011	28	KYO64538
	Aspergillus flavus AF70	oxidoreductase, short chain dehydrogenase/reductase family	45.4	20	0.13	29	KOC18052
	Amycolatopsis keratiniphila	nitroreductase	43.5	11	0.14	36	WP_063275982
	Amycolatopsis orientalis	nitroreductase	43.5	11	0.14	36	WP_046199795
	Aspergillus oryzae RIB40	oxidoreductase, short chain dehydrogenase/reductase family	44.7	20	0.26	29	XP_001727173
	Amycolatopsis orientalis HCCB10007	hypothetical protein AORI_3756	42.7	11	0.29	36	AGM06341
	Labrenzia sp. DG1229	methyltransferase type 11	43.5	18	0.34	28	WP_029061648
	Nocardia niwae	hypothetical protein	43.9	18	0.35	28	WP_063019054
	Amycolatopsis azurea	nitroreductase	41.6	11	0.62	33	WP_052014240
	Desulfuromonas acetoxidans	SAM-dependent methyltransferase	43.9	27	0.82	22	WP_005999076
	Mycobacterium kansasii	6-O-methylguanine DNA methyltransferase	42	23	1.1	29	KZS84804
	Amycolatopsis nigrescens	hypothetical protein	41.2	9	1.1	36	WP_020673584
	Amycolatopsis jejuensis	nitroreductase	40.8	11	1.1	37	WP_033287732
	Amycolatopsis sp. MJM2582	nitroreductase	40.8	11	1.1	32	WP_052120702
4	Cladosporium cladosporioides virus 1	hypothetical protein	180	86	7E-52	40	YP_009052473
	Aspergillus fumigatus tetramycovirus-1	PAS-rich protein	144	81	7E-38	41	CDP74621
	Botryosphaeria dothidea virus 1	hypothetical protein	143	83	1E-37	38	AKE49498
	Cyanothece sp. CCY0110	hypothetical protein	39.7	15	1.1	35	WP_008275724
5-1	Drosophila melanogaster	short stop, isoform K	40	36	4.3	27	NP_001137659
	Drosophila melanogaster	short stop, isoform G	40	36	4.3	27	NP_725335
	Drosophila yakuba	uncharacterized protein Dyak_GE13333, isoform AH	39.7	36	4.4	27	XP_015052457
5-2	no hits	/	/	/	/	/	/
6	Thiomonas sp. CB2	conserved hypothetical protein	37	42	7.3	32	CDW93413
7	Olsenella sp. oral taxon 807	transketolase	38.1	37	4.9	32	WP_050341181
8-1	no hits	/	/	/	/	/	/
8-2	no hits	/	/	/	/	/	/

Supplementary Table 3 Predicted homology proteins by HHpred tool with the ones deduced on the basis of open reading frames (ORFs) of dsRNAs 1 to 8 of Colletotrichum camelliae filamentous virus 1 (CcFV-1).

**No.**	**Hit**	**Homologyprotein**	**Host**	**Prob (%)**	**E-value**	**Score**	**Query**	**Iden(%)**	**Simi ***
1	3uqs_A	RNA-dependent RNA polymerase; transferase	*Murine norovirus* (Caliciviridae)	100.0	$5.9E^{-50}$	457.1	211-695	14.0	0.310
	2ckw_A	RNA-directed RNA polymerase; hydrolase, nucleotide-binding, nucleotidyltransferase, covalent protein-RNA linkage, RNA elongation	*Sapporo virus* (Caliciviridae)	100.0	$3.3E^{-49}$	450.0	211-688	17.0	0.187
	4zpc_A	RNA-dependent RNA polymerase; transcription	*Coxsackievirus* B3 (Picornaviridae)	100.0	$1.9E^{-48}$	438.6	210-652	18.0	0.189
	3bso_A	RNA dependent RNA polymerase; RNA-dependent RNA polymerase, viral replication, antiviral E inhibitor, helicase, hydrolase	*Norwalk virus* (Caliciviridae)	100.0	$4.0E^{-48}$	440.9	211-688	15.0	0.159
	1khv_A	RNA-directed RNA polymerase; RNA-dependent RNA polymerase, transferase	*Rabbit hemorrhagic disease virus*(Caliciviridae)	100.0	$1.3E^{-47}$	436.9	211-686	16.0	0.111
	4wyw_A	RNA-directed RNA polymerase 3D-POL; closed-right hand RNA dependent RNA polymerase picornavirus, transferase	*Foot-and-mouth disease virus*(Picornaviridae)	100.0	$2.1E^{-47}$	432.2	211-686	17.0	0.181
	1xr7_A	Genome polyprotein; RNA-dependent RNA polymerase, transferase	*Human rhinovirus* 16(Picornaviridae)	100.0	$1.0E^{-45}$	415.6	210-652	19.0	0.249
	2ijd_1	RNA-directed RNA polymerase; RNA-dependent RNA polymerase, picornavirus, protease, hydrolase, transferase	*Human poliovirus* 1(Picornaviridae)	100.0	$8.3E^{-46}$	433.2	210-655	18.0	0.169
	5f8h_A	Genome polyprotein; polymerase-RNA complex, elongation, nucleotide addition CYCL transferase-RNA complex	*Enterovirus* A71(Picornaviridae)	100.0	$6.6E^{-41}$	377.4	211-655	17.0	0.197
	4nyz_A	Genome polyprotein; encephalomyocarditis virus, close right hand, RNA dependent polymerase, transferase	*Mengo virus*(Picornaviridae)	100.0	$4.0E^{-40}$	369.3	211-677	15.0	0.123
2	2kc5_A	Hydrogenase-2 operon protein HYBE; chaperone	*Escherichia coli*	23.0	29.0	33.1	91-200	30.0	0.501
	1j26_A	Immature colon carcinoma transcript 1; peptide chain release factors, RF-1, the GGQ motif, immature carcinoma transcript 1	*Mus musculus*	21.4	60.0	29.2	354-391	16.0	0.320

	2jva_A	Peptidyl-tRNA hydrolase domain protein; GFT hydrolase, structural genomics, PSI-2, protein STRU initiative	*Pseudomonas syringae* PV	20.2	87.0	28.0	353-397	11.0	0.163
3	1xxl_A	YCGJ protein; structural genomics, protein structure initiative, PSI, NEW YORK SGX research center for structural genomics, nysgxrc	*Bacillus subtilis*	99.8	$1.40E^{-17}$	166.1	113-309	21 .0	0.197
	3dlc_A	Putative S-adenosyl-L-methionine-dependent methyltransferase; structural genomics, joint center for structural genomics	*Methanococcus maripaludis*	99.7	$5.0E^{-17}$	156.7	112-308	13.0	0.097
	1vl5_A	Unknown conserved protein BH2331; putative methyltransferase, structural genomics, joint cente structural genomics, JCSG	*Bacillus halodurans*	99.7	$3.0E^{-16}$	157.6	116-310	16.0	0.215
	5fcd_A	MCCD, MCCD protein; methyltransferase fold, SAM binding, MCC bindi structural genomics, center for structural genomics of INFE diseases, csgid	*Escherichia coli*	99.7	$3.70E^{-16}$	156.9	113-309	11.0	0.019
	3ujc_A	Phosphoethanolamine N-methyltransferase; parasite	*Plasmodium falciparum*	99.7	$8.0E^{-17}$	163.0	114-308	14.0	0.124
	2yqz_A	Hypothetical protein TTHA0223; RNA methyltransferase, SAM, structural genomics, NPPSFA	*Thermus thermophiles*	99.7	$9.6E^{-16}$	155.0	112-351	14.0	0.059
	5egp_A	UBIE/COQ5 family methyltransferase, putative; BIS-thiomethyltransferase, gliotoxin, epipolythiodioxopipera aspergillus fumigatus	*Aspergillus fumigatus* Z5	99.7	$5.6E^{-16}$	159.8	112-326	11.0	0.036
	3bus_A	REBM, methyltransferase; rebeccamycin synthesis	*Lechevalieria aerocolonigenes*	99.7	$9.2E^{-16}$	155.4	113-308	17.0	0.151
	3ccf_A	Cyclopropane-fatty-acyl-phospholipid synthase; YP_321342.1, putative methyltransferase	*Anabaena variabilis* atcc 29413	99.7	$1.9E^{-15}$	153.6	118-407	12.0	0.031
	4kdc_A	3-demethylubiquinone-9 3-methyltransferase; rossmann fold, O-methylation, ubiquinone biosynthesis	*Escherichia coli*	99.67	$1.3\ E^{--15}$	151.7	113-314	17.0	0.125
4	1b4u_A	LIGA, LIGB, protocatechuate 4,5-dioxygenase; extradiol type dioxygenase, non-heme iron protein	*Sphingomonas paucimobilis*	66.6	3.1	35.1	74-97	13.0	0.304
	2ll2_A	GAP junction alpha-1 protein; membrane protein	*Homo sapiens*	58.0	2.8	27.4	210-217	63 .0	1.228
	4wj3_C	Glutamyl-tRNA(Gln) amidotransferase subunit C; transamidosome, aminoacyl-tRNA synthetase, gatcab, LIG complex	*Pseudomonas aeruginosa* PAO1	55.2	9.4	29.2	17-51	23 .0	0.257
	3ip4_C	Aspartyl/glutamyl-tRNA(Asn/Gln) amidotransferase; multi protein complex, ligase, ATP-binding, nucleotide-binding protein biosynthesis	*Staphylococcus aureus* subsp	54.2	13.0	27.8	19-51	18.0	0.301
	3kfu_G	Glutamyl-tRNA(Gln) amidotransferase subunit C; ASPRS,	*Thermus thermophilus*	52.9	14.0	27.2	19-51	18.0	0.279

		gatcab, ATP-binding, aminoacyl-tRNA synthetase, ligase nucleotide-binding, protein biosynthesis, ligase-RNA complete							
	1d2n_A	N-ethylmaleimide-sensitive fusion protein; hexamerization domain, ATPase, transport	*Cricetulus griseus*	51.1	3.6	32.8	70-139	13.0	0.113
	3wra_A	Gallate dioxygenase; oxidoreductase, extradiol dioxygenase, Fe2+ binding, domain- dimer	*Sphingomonas paucimobilis*	46.8	7.9	35.9	74-97	21.0	0.427
	3h0l_C	Glutamyl-tRNA(Gln) amidotransferase subunit C; multi protein complex, ligase, protein biosynthesis	*Aquifex aeolicus*	40.0	31.0	25.4	21-51	23.0	0.371
	3ur8_A	Glucan endo-1,3-beta-D-glucosidase; glucoside hydrolase, GH17 family, pathogenesis-related class protein (PR-2), TIM barrel	*Solanum tuberosum*	39.9	70.0	26.2	108-186	17.0	0.332
	1aq0_A	1,3-1,4-beta-glucanase; hydrolase, glycosidase, glycoprotein, glycosylated protein	*Hordeum vulgare*	34.3	61.0	26.3	114-186	24.0	0.342
	2b7t_A	Double-stranded RNA-specific editase 1; RNA editing, RNA-binding protein, hydrolase	*Rattus norvegicus*	24.3	160.0	35.1	168-206	24.0	0.248
5-1	2l0d_A	Cell surface protein	*Methanosarcina acetivorans*	38.4	100 .0	20.5	200-254	19.0	0.315
	3iym_A	Capsid protein; dsRNA virus, icosahedral virus, partitivirus, PSV-S	*Penicillium stoloniferum virus* S	32.5	12.0	35.8	112-133	50.0	0.75
5-2	5ewn_A	Structural protein; virus protein, capsid protein, icosahedral virus, jelly-roll protein	*Human astrovirus*-1	45.3	7.2.0	27.4	28-35	63.0	1.830
	3nut_A	Precorrin-3 methylase; vitamin B12 pathway, cobalamin, methyltransferase, transfera	*Rhodobacter capsulatus*	34.4	17.0	20.6	13-38	22.0	0.463
	1t8h_A	YLMD protein sequence homologue; rbstp0554	*Geobacillus stearothermophilus*	33.7	12.0	23.5	3-8	100.0	1.752
	3hag_A	Capsid protein; jelly-roll beta sheets, beta barrel, icosahedral virus	*Hepatitis e virus* type 4	33.7	13.0	27.3	28-35	38.0	1.016
	1xfj_A	Conserved hypothetical protein; HYP protein, alpha-beta-BETA-alpha	*Caulobacter vibrioides*	33.3	10.0	23.5	3-8	83.0	1.486
	1rv9_A	Conserved hypothetical protein NMB0706; alpha-beta-BETA-alpha structure	*Neisseria meningitidis*	31.7	11.0	23.7	3-8	83.0	1.298
	1z9t_A	Hypothetical UPF0124 protein YFIH; putative laccase	*Escherichia coli*	30.8	12.0	23.4	3-8	83.0	1.298
	2mxb_A	EPO-R, erythropoietin receptor; membrane protein	*Mus musculus*	28.4	6.8	21.6	3-10	63.0	0.974
6	4j8q_A	Putative uncharacterized protein; NIGD-like protein, PF12667 family	*Bacteroides fragilis*	61.4	4.6	30.5	145-176	22.0	0.232

	3k6o_A	Uncharacterized protein DUF1344; protein of unknown function DUF1344	*Bacteroides vulgatus* atcc 8482	59.7	4.0	30.7	143-173	29.0	0.393
	3qwn_A	Hypothetical NIGD-like protein; SH3-like barrel fold	*Bacteroides caccae*	41.5	8.7	29.4	139-166	14.0	0.176
	2fhw_B	Relaxin 3 (prorelaxin H3) (insulin-like peptide INSL7) (insulin-like peptide 7); insulin/relaxin super-family fold, signaling protein	Synthetic	41.3	19.0	21.5	14-26	46.0	0.953
	4pqx_A	Uncharacterized protein; NIGD-like protein, PF12667 family	*Bacteroides caccae*	38.4	13.0	28.5	155-183	34.0	0.502
	2h8b_B	Insulin-like 3; insulin/relaxin suparfamily fold, hormone/growth factor complex	Synthetic	37.6	22.0	21.7	14-26	46.0	1.025
	6rlx_B	Relaxin, B-chain; hormone(muscle relaxant)	*Homo sapiens*	29.8	19.0	21.5	14-26	38.0	0.600
	4c5i_C	Transcriptional repressor protein YY1	*Homo sapiens*	28.6	25.0	21.7	23-30	50.0	1.315
	3k0y_A	Putative toxin related protein	*Parabacteroides distasonis* atcc 8503	23.2	30.0	26.9	146-171	38.0	0.539
	2kbc_B	INSL5_B-chain; peptide hormone, relaxin, insulin-like fold	Synthetic	22.6	21.0	20.9	11-23	31.0	0.567
7	2raa_A	Pyruvate synthase subunit PORC; TM0015, pyruvate oxidoreductase subunit PORC (EC 1.2.7.1), S genomics	*Thermotoga maritima* MSB8	37.1	3.8	30.7	122-154	30.0	0.420
	3g2e_A	OORC subunit of 2-oxoglutarate:acceptor oxidoreductase	*Campylobacter jejuni*	34.4	4.0	30.0	122-154	30.0	0.379
	1j6q_A	Cytochrome C maturation protein E; all-beta protein, heme delivery,cytochrome C maturation, OB-(oligonucleotide binding)fold	*Shewanella putrefaciens*	30.0	10.0	28.6	128-156	28.0	0.630
	1sr3_A	APO-CCME; OB fold, beta barrel, flexible C-terminal domain, chaperone	*Escherichia coli*	28.0	13.0	28.2	128-156	31.0	0.547
	2ws9_1	P1	*Equine rhinitis a virus*	25.0	68.0	24.6	40-100	22.0	0.416
	2he9_A	NK-tumor recognition protein; cyclosporin, isomerase, membrane, repeat, rotamase, peptidylprolyl isomerase	*Homo sapiens*	24.7	70.0	24.0	105-149	20.0	0.256
	3on3_A	Keto/oxoacid ferredoxin oxidoreductase, gamma SUB	*Geobacter sulfurreducens*	24.4	4.0	29.7	122-154	24.0	0.449
	5cfc_A	Genome polyprotein,VP1; virion, capsid, pathogen	*Saffold virus*	21.2	78.0	24.8	40-100	17.0	0.544
	4i9y_A	E3 SUMO-protein ligase ranbp2; nuclear pore complex, transport protein	*Homo sapiens*	21.0	130.0	21.8	110-149	18.0	0.227
	1tme_1	Chain 1, Three-Dimensional Structure Of Theiler Virus	murine encephalomyelitis virus	20.3	100.0	24.9	40-100	19.0	0.491
8-1	3ie4_A	GRAM-negative binding protein 3; immunoglobulin fold, immune system	*Drosophila melanogaster*	32.5	2.0	25.1	56-105	26.0	0.460
	2f6k_A	Metal-dependent hydrolase; metal dependent hydrolyse, aminohydro_2, ACMDS, ACMS, trypto metabolism,	*Lactobacillus plantarum*	30.5	14.0	27.4	29-47	22.0	0.416

		quinolinic acid, QUIN							
	3aqy_A	Beta-1,3-glucan-binding protein; beta-sandwich, immune receptor, sugar bindi protein	*Plodia interpunctella*	29.3	24.0	24.8	79-105	30.0	0.708
	2kha_A	Beta-1,3-glucan-binding protein; glycoprotein, immune response, innate immunity, SEC sugar binding protein	*Plodia interpunctella*	26.7	37.0	25.3	79-105	33.0	0.736
	4dm5_A	Osmotically inducible lipoprotein OSME; unknown function	*Pseudomonas aeruginosa* PAO1	26.0	39.0	23.8	52-119	17.0	0.158
	2hbv_A	2-amino-3-carboxymuconate 6-semialdehyde decarbox; ACMSD, TIM-barrel, decarboxylase, metaloenzyme, lyase	*Pseudomonas fluorescens*	24.3	29.0	25.9	29-47	50.0	0.802
	2gfg_A	BH2851; antiparallel barrel, structural genomics, joint center for S genomics, JCSG, protein structure initiative, PSI-2, unknow function	*Bacillus halodurans*	23.9	78.0	21.7	32-60	24.0	0.310
	2krc_A	DNA-directed RNA polymerase subunit delta; delta subunit, GRAM-positive bacteria, nucleotidyltransferase, transcription, transferase	*Bacillus subtilis*	23.7	20.0	25.4	59-78	40.0	0.883
	3ij6_A	Uncharacterized metal-dependent hydrolase; structural genomics, amidohydrolase, PSI-2, protein structure	*Lactobacillus acidophilus*	23.5	31.0	25.5	29-47	33.0	0.676
	4do7_A	Amidohydrolase 2; enzyme function initiative, EFI, structural TIM-barrel fold, putative lactonase	*Burkholderia multivorans*	22.7	31.0	25	31-47	50.0	0.922
	2lxt_C	Cyclic AMP-responsive element-binding protein 1; CREB binding protein	*Homo sapiens*	22.4	27.0	22.2	123-137	47.0	0.769
	4ofc_A	2-amino-3-carboxymuconate-6-semialdehyde decarbox; TIM-barrel, decarboxylase, oxidoreductase	*Homo sapiens*	20.4	38.0	25.3	29-47	39.0	0.641
8-2	4nar_A	Putative uronate isomerase	*Thermotoga maritima*	48.6	8.7	25.9	6-32	35.0	0.386
	3tmm_A	Transcription factor A, mitochondrial; HMG, high mobility group, transcription, LSP1, mitochon transcription-DNA complex	*Homo sapiens*	38.7	8.3	23	27-47	38.0	0.631
	2zaj_A	Membrane-associated guanylate kinase, WW and PDZ domain-containing protein 1; BAI1-associated protein 1 (BAP-1)	*Homo sapiens*	30.3	13.0	16.6	28-44	41.0	0.585
	3oun_A	Putative uncharacterized protein TB39.8; peptidoglycan, Ser/Thr kinase, pseudokinase, FHA domain, REG phosphorylation	*Mycobacterium tuberculosis*	30.2	14.0	21.6	27-47	38.0	0.637
	5ctq_A	Squamous cell carcinoma antigen recognized by T-C; half A tetratricopeptide repeat (HAT) domain, protein transp immune system, nuclear protein	*Homo sapiens*	27.2	17.0	22.1	28-46	42.0	0.695

	2dal_A	Protein KIAA0794; FAS associted factor 1, UBA-like domain, structural genomics, NPPSFA	*Homo sapiens*	27.0	16.0	17.7	28-34	71.0	0.928
	2plj_A	Lysine/ornithine decarboxylase; type IV decarboxylase, beta/alpha barrel, beta barrel, lyase	*Vibrio vulnificus*	26.8	17.0	23.4	27-46	40.0	0.702
	4xvc_A	Esterase E40; hydrolase	*Environmental samples*	21.3	27.0	20.0	27-47	38.0	0.631

*No, Index of deduced protein from ORFs 1 to 8-2 of dsRNAs 1 to 8, respectively; Hit, domain ID and beginning of description of the matched database domain; Homology protein, Predicted homology proteins by HHpred tool with the inquiry; Host, from which the predicted proteins derived; Prob, Probability of template to be a true positive; E-value: Expect-value; Score, Raw score. The raw score is calculated by comparing the amino acid distributions between columns from the query alignment and columns from the template alignment; Query, Range of query match states aligned.

Supplementary Table 4 Virus names, GenBank accession numbers, taxonomic statuses, genomic numbers, and virion morphologies of the isolates used for the RdRp phylogenetic analysis shown in Supplementary Fig. 2.

Virus name	Abbreviation	GenBank Acc.no.	Genus*	Family**	Genome***	Structure
Colletotrichum camelliae filamentous virus 1	CcFV-1	KX778766	/	/	8 ds	Filamentous
Aspergillus fumigatus tetramycovirus-1	AFuTmV-1	CDP74618	/	/	4 ds	non-particle
Beauveria bassiana polymycovirus-1	BbPmV-1	LN896307	/	/	4 ds	non-particle
Beauveria bassiana polymycovirus-2	BbPmV-2	LN896318	/	/	7 ds	non-particle
Botryosphaeria dothidea virus 1	BdRV1	KP245734	/****	/	5 ds	unknown
White clover cryptic virus 1	WccV	AAU14889	*Alphapartiti-*	*Partiti-*	2 ds	Icosahedral
Penicillium stoloniferum virus S	PsV-S	YP_052857	*Gammapartiti-*	*Partiti-*	2 ds	Icosahedral
Citrullus lanatus cryptic virus	CLCV	AGH12859	*Deltapartiti-*	*Partiti-*	2 ds	Icosahedral
Black raspberry cryptic virus	BRCV	ABU55400	*Deltapartiti-*	*Partiti-*	2 ds	Icosahedral
Persimmon cryptic virus	PeCV	CCH50609	*Deltapartiti-*	*Partiti-*	2 ds	Icosahedral
Mycoreovirus 1	MyRV1	AAP45577	*Mycoreo-*	*Reo-*	10-12 ds	double-shelled
Mycoreovirus 3	MyRV3	YP_392478	*Mycoreo-*	*Reo-*	10-12 ds	double-shelled
Saccharomyces cerevisiae virus L-A	ScV-L-A	AAA50321	*Toti-*	*Toti-*	1 ds	Icosahedral
Helminthosporium victoriae 190S virus	Hv190SV	O57044	*Victori-*	*Toti-*	1 ds	Icosahedral
Penicillium chrysogenum virus	PcV	YP_392482	*Chryso-*	*Chryso-*	4 ds	Icosahedral
Verticillium chrysogenum virus	VcV	ADG21213	*Chryso-*	*Chryso-*	4 ds	Icosahedral
Rosellinia necatrix quadrivirus 1	RnQV1	BAM93353	*Quadri-*	*Quadri-*	4 ds	Icosahedral
Rosellinia necatrix megabirnavirus 1	RnMBV1	YP_003288763	*Megabirna-*	*Megabirna -*	2 ds	Icosahedral
Sclerotinia sclerotiorum megabirnavirus 1	SsMBV1	YP_009143529	*Megabirna-*	*Megabirna -*	2 ds	Icosahedral
Phytophthora endornavirus 1	PEV1	YP_241110	*Endorna-*	*Endorna-*	1 ds	non-particle
Helicobasidium mompa endornavirus 1	HmEV1	YP_003280846	*Endorna-*	*Endorna-*	1 ds	non-particle
Human picobirnavirus	HPV	AHX00960	*Picobirna-*	*Picobirna-*	2 ds	Icosahedral
Norovirus	NV	AJT43260	*Noro-*	*Calici-*	1 +ss	Icosahedral
Rabbit hemorrhagic disease virus	RHDV	NP_740332	*Lago-*	*Calici-*	1 +ss	Icosahedral
Murine norovirus 1	MNV-1	YP_720001	*Noro-*	*Calici-*	1 +ss	Icosahedral
Turkey avisivirus	TuASV	AGH06056	*Avisiviru*	*Picorna-*	1 +ss	Icosahedral
Duck hepatitis A virus	DHAV	AHJ78642	*Avihepato-*	*Picorna-*	1 +ss	Icosahedral
Avian nephritis virus 2	ANV-2	ADQ43328	*Avastro-*	*Astro-*	1 +ss	Icosahedral
Duck astrovirus 1	DAstV1	ADB79803	*Avastro-*	*Astro-*	1 +ss	Icosahedral

* and **,'-'refer to omitted'virus'and 'viridae', respectively; ***, ds, double stranded RNA, +ss, positive single stranded RNA;****, the taxonomic status is unclear.

Supplementary Table 5 Summary of the peptide mass fingerprinting analysis of p80 and p70.

Species	Protein	Calculated Mass	Observed Mass	± delta	Amino acid sequence	Ion score	GenBank accession no.
Colletotrichum gloeosporioides *	glucoamylase	1119.6288	1120.644	1.0152	VVTQFGQTIK	27	gi|596650956
		1445.6616	1446.6581	0.9965	TDPPYFYTWTR	59	
		1736.8733	1737.8754	1.0021	NGYSSTASSVLWPVIR	120	
		1782.9265	1783.9215	0.9950	QQGSITVTQTSLAFFR	110	
		1932.0428	1933.0355	0.9927	TIYTINNGIAQSAAIAVGR	125	
		1967.8948	1968.8949	1.0001	FWSPSGNFMVANINGNGR	84	
		2582.2401	2583.2085	0.9684	SNGQPLSADDLTWSYAAFLTAA QR	153	
		3028.489	3029.438	0.9490	AVVDIFTNSYDANLQTNIQNYI ASQAR	233	
Colletotrichum higginsianum	glycosyl hydrolase family 15	1782.9265	1783.9215	0.9950	QQGSITVTQTSLAFFR	110	gi|380482789
		1932.0316	1933.0355	1.0039	TIYVINNGIAQSAAIAVGR	85	
		2582.2401	2583.2085	0.9684	SNGQPLSADDLTWSYAAFLTAA QR	153	
Colletotrichum gloeosporioides	hypothetical protein CGLO_02068	1049.5182	1050.5236	1.0054	VFDEWNIK	44	gi|530478401
		1465.7202	1466.7201	0.9999	AKPGVESFDDFVR	69	
		1795.9006	1796.8912	0.9906	VPNYHAPGEFIQPTAR	106	
		3076.5658	3077.521	0.9552	AAGVEPIVDLPGVGLNFQDHYL TFSVYR	73	
Colletotrichum gloeosporioides **	calpain family cysteine protease	1341.6313	1342.6388	1.0075	NQTGSDALYFAC	94	gi|530474949
		1401.6425	1402.6448	1.0023	GSEWYNPGSIHV	62	
		1470.8042	1471.8074	1.0032	AGTAVIVLTQLDDY	97	
		1571.842	1572.84	0.9980	SGLALGHAYSILQSE	86	
		1598.8992	1599.9005	1.0013	KAGTAVIVLTQLDDY	56	
		1777.7544	1778.7506	0.9962	SESGVGEWNGPWSDGSE	96	
		2418.138	2419.1331	0.9951	VDWIFESPQFTVNGYSSSDIQ	49	
		2654.2475	2655.2388	0.9913	ELVNADGEFVFALAAMGTGW DWQS	30	
		3276.4994	3277.4429	0.9435	VHGDYEAISGGWPGEAVEDMT GGVTSTIATNV	190	
Colletotrichum gloeosporioides	calpain-like protein	1341.6313	1342.6388	1.0075	NQTGSDALYFAC	94	gi|596659795
		1470.8042	1471.8074	1.0032	AGTAVIVLTQLDDY	97	
		1571.842	1572.84	0.9980	SGLALGHAYSILQSE	86	
		1598.8992	1599.9005	1.0013	KAGTAVIVLTQLDDY	56	
		2003.9781	2004.9677	0.9896	GFEGQYYFELHFVLQT	53	
		3276.4994	3277.4429	0.9435	VHGDYEAISGGWPGEAVEDMT GGVTSTIATNV	190	
Colletotrichum gloeosporioides	glucoamylase	1735.8734	1736.8807	1.0073	NGYSSTASSVLWPVIN	51	gi|596650956
		1933.0216	1934.0289	1.0073	TIYTINNGIAQSAAIAVGY	77	
		1968.8777	1969.885	1.0073	FWSPSGNFMVANINGNGS	47	
		2582.197	2583.2043	1.0073	SNGQPLSADDLTWSYAAFLTAA QH	130	
		3028.4258	3029.4331	1.0073	AVVDIFTNSYDANLQTNIQNYI ASQAL	96	
Colletotrichum salicis	calpain family cysteine protease	1341.6565	1342.6388	0.9823	LQTGSDALYFAC	77	gi|996612876
		1571.842	1572.84	0.9980	SGLALGHAYSILQSR	86	

* and **, the peptided match p80 (above panel) and p70 (below panel), respectively.

Supplementary Table 6 Summary of the peptide mass fingerprinting analysis of p31 encoded by ORF4 of CcFV-1 dsRNA4.

Amino acid position	Calculated Mass	Observed Mass	± delta	Amino acid sequence	Ion score
2-12	1174.5506	1175.5690	-1.0184	SSSASYSFLGK	46
18-31	1452.8552	1453.8771	-1.0219	VAALDQGVVELVIK	107
37-53	1769.0087	1770.0342	-1.0255	LKPPALLAYATEVAQGK	48
54-79	2754.4817	2755.4927	-1.0110	VPTAALSVGTPKPVTIQAHSWLNDPR	31
54-79	2786.4715	2787.4915	-1.0200	VPTAALSVGTPKPVTIQAHSWLNDPR	26
85-93	1052.4774	1053.4955	-1.0181	YGVSEEDVR	80
110-119	1133.6445	1134.6648	-1.0203	ISAIVTDFLR	82
110-120	1261.7394	1262.7578	-1.0184	ISAIVTDFLRK	35
129-149	1980.9865	1982.0178	-1.0313	VVSTGDPGTNPATANPLAGSR	104
150-160	1164.6139	1165.6340	-1.0201	GLVASYASEIR	95
161-178	2000.8752	2001.9031	-1.0279	SNSGVYGSYTFETFETGR	125
179-199	2275.1485	2276.1777	-1.0292	DVTQAFGVDLGHEVYVLAQSK	68
215-231	1884.9621	1885.9900	-1.0279	TDPLALPYIYYISSGGR	105
Note: The overall protein score is 692.					

Supplementary Table 7 Families and genera of filamentous viruses and dsRNA viruses listed according to the nature of the genome presented in the 9th International Committee on Taxonomy of Viruses (ICTV) [2].

Family or unassigned genus	Nature of the genome	Morphology	Virion size (nm)	Genome configuration	Genome size (kbp or kb)	Host	Group
Lipothrixviridae	dsDNA	filamentous	24-38×410-2200	1 linear segment	16-42	Ar	I
Inoviridae; inoviruse	ssDNA(+)	filamentous	7×700-3500	circular genome	5.8-12.4	B	II
Ophioviridae	ssRNA (-)	filamentous, coiled	3×≥760	3 or 4 linear segments	7.6-8.2, 1.6-1.8, 1.5, 1.4	P	V
Tenuivirus	ssRNA (+/-)	filamentous	3-10×>100	4-6 linear segments	total genome: 17-23	P, I	V
Alphaflexiviridae	ssRNA (+)	filamentous	10-15 × 470-800	1 linear segment	6-9	P,F	IV
Betaflexiviridae	ssRNA (+)	filamentous	10-15 ×600-1000	1 linear segment	6-9	P	IV
Gammaflexiviridae	ssRNA (+)	filamentous	13×720	1 linear segment	7	F	IV
Closteroviridae	ssRNA (+)	filamentous	12× 650-2000	1-3 linear segments	monopartite:13-19; bipartite: 8-9, tripartite: 8, 5.3, 3.9	P	IV
Potyviridae	ssRNA (+)	filamentous	monopartite:11-15 ×650-900; bipartite: 11-15 ×250-300 and 500-600	1 or 2 linear segments	monopartite: 9.3-10.8; bipartite: 7.3-7.6, 3.5-3.7	P	IV
Birnaviridae	dsRNA	icosahedral	65	2 linear segments	3.5, 3	V, I	III
Chrysoviridae	dsRNA	icosahedral	35-40	4 linear segments	3.6, 3.2, 3.0, 2.9	F	III
Cystoviridae	dsRNA	spherical	85	3 linear segments	6.4-7.1, 3.6-4.7, 2.6-3.2	B	III
Endornaviridae	dsRNA	pleomorphic RNA-containing vesicles	no virions; viral RNA contained in cytoplasmic vesicles	1 linear segment	14-18	P, F, O	III
Partitiviridae	dsRNA	icosahedral	30-43	2 linear segments	1.4-2.4 per segment	P, F	III
Picobirnaviridae	dsRNA	icosahedral	33-37	2 linear segments	2.4-2.6, 1.5-1.9	V	III
Reoviridae	dsRNA	turreted or non-turreted icosahedral capsid	60-85	9-12 linear segments	total genome: 19-32	F, I, P, V, Pr	III
Totiviridae	dsRNA	icosahedral	33-40	1 linear segment	4-7	F, Pr	III

Abbreviations of the virus hosts: Algae, Al; Archaea, Ar; Bacteria, B; Fungi, F; Invertebrates, I; Plants, P; Protozoa, Pr; Vertebrates, V; Oomycete, O.

Supplementary References

1 Wang, L. *et al*. Hypovirulence of the phytopathogenic fungus *Botryosphaeria dothidea*: Association with a coinfecting chrysovirus and a partitivirus. *J. Virol.* **88**, 7517-7527 (2014).

2 Abergel, C. *et al*. *Virus Taxonomy: Ninth Report of the International Committee on Taxonomy of Viruses*. 23-1196 (Elsevier Academic Press, 2011).
